# Spatial pattern of Bois noir: case study of a delicate balance between disease progression and recovery

**DOI:** 10.1038/s41598-020-66210-7

**Published:** 2020-06-17

**Authors:** Sergio Murolo, Matteo Garbarino, Valeria Mancini, Gianfranco Romanazzi

**Affiliations:** 1 Department of Agricultural, Food and Environmental Science, Marche Polytechnic University, Ancona, Italy; 2 Department of Agricultural, Forest and Food Sciences, University of Turin, Turin, Italy

**Keywords:** Ecological epidemiology, Biotic

## Abstract

Bois noir (BN) is the most important phytoplasma disease of Euro-Mediterranean area and induces severe loss of production and even the death of vines. Understanding the delicate balance between disease progression and recovery of BN infected plants over space and time is crucial to set up management tools. The data collected and analysed allowed to provide insights into dispersal pattern of the disease, caused by’*Candidatus* Phytoplasma solani’. Point pattern analysis (PPA) was applied to assess the spatial arrangement of symptomatic plants and the spatial correlation of disease levels in four vineyards. For spatio-temporal patterns of BN, a mark-correlation function was applied. Space-time PPA over multiple years (2011–2015) provided graphical visualisation of grapevines more severely affected by BN along the borders of the vineyards, mainly in 2011 when disease incidence was high. PPA across the symptomatic plants in the four vineyards confirmed this visual trend: an overall aggregated pattern at small (<10 m) spatial scales (2013) that were more evident later at all spatial scales (0–15 m). Application of this innovative spatial approach based on point and surface pattern analyses allowed the spread and severity of BN to be monitored, to define the dispersal routes of the pathogen. Such data will contribute to better understand the distribution of symptomatic plants over space and time and to define a model for preventive strategies to reduce future infections.

## Introduction

Bois noir (BN) is one of the most important grapevine yellows in Europe^1^ and phytoplasma related to ‘stolbur group’ are today emergent threats for the grapevine cultivation and for vegetable crops in South and East Asia^2,3^. BN is generally considered less epidemic than Flavescence dorée (FD), although it is more difficult to manage, because no direct means are available for the pathogen and the main vectors^4^. Over recent decades, frequent BN outbreaks have been recorded in the main viticultural areas^3,5–9^, which have led to dramatic losses in grape quality and quantity, most of all for highly susceptible grapevine cultivars such as ‘Chardonnay’^1,10,11^.

The causal agent of BN is a phytoplasma that belongs to the stolbur group (16SrXII-A subgroup) and has been assigned to ‘*Candidatus* Phytoplasma solani’ (‘*Ca*. P. solani’)^12^. It is transmitted mainly by the polyphagous planthopper *Hyalesthes obsoletus* (Sign., 1865, Hemiptera: Cixiidae) to a wide range of wild plants, such as *Convolvulus arvensis*, *Calystegia sepium*, *Urtica dioica*^13^. However, several weeds were reported as hosts of ‘*Ca*. P. solani’ within and around infected vineyards and can therefore play a key role in the BN spreading^8,14–18^.

Thus, all of these hosts represent potential inoculum sources, while for grapevines, the occasional infections by *H. obsoletus* represent dead-end hosts for the stolbur phytoplasma.

BN symptoms include abnormal lignification of canes, short internodes, flower abortion, and curling and discoloration of leaves, with intervein yellowing or reddening^1,10^. These are consequences of modifications to the plant physiology caused by ‘*Ca*. P. solani’ infection, as perturbations to leaf gas exchange, fluorescence of chlorophyll a, pigment content and maximum quantum efficiency of photosystem II^10^. These modifications are deeply influenced by alterations in the expression of genes in infected grapevines^19–21^.

An interesting but still not completely understood aspect of phytoplasma–plant interactions is the process known as ‘recovery’, where there is spontaneous disappearance of symptoms in symptomatic plants, which is then in most cases (~80%) permanent^22–24^. In ‘recovered’ grapevines, ‘*Ca*. P. solani’ has been recently detected in the roots^25^, although it has not been recorded from the canopy^10,24,26^, and the production can be intermediate between the symptomatic and healthy grapevines especially in the first years, performing to healthy grapevines later^10,22,24,27,28^.

The incidence and economic impact of BN on grapevine production are determined by the equilibrium of the rates of new and retained symptomatic grapevines, remission and re-occurrence of symptoms, combined with the complete recovery of previously infected grapevines, and dead plants^4,23^.

Spatial pattern analysis has undergone rapid expansion in plant-disease epidemiology, and it represents an instrument that can lead to improved disease-management strategies^29–31^. Recently, several studies have focused on spatial analysis, to improve knowledge about BN epidemiology, and the possible roles of the host plants and the insect vectors in the spread of such phytoplasma investigated^6,14,32^.

Here, we focused on BN and cv ‘Chardonnay’, as a model-type for studying the spatial distribution of symptomatic plants, to provide insights about dispersal pattern of such disease, how the disease severity of plants can change in space and in time, how much the recovered plants can show again symptoms and how much the symptomatic plants can to recover.

This study was designed on the following lines: (i) to record the modulated intensity and severity of BN and the recovery phenomenon in a pilot vineyard located in Montalto delle Marche (MOV) over a time-span of 7 years; (ii) to analyse the epidemiological pattern of BN in the pilot vineyard (MOV), and in a further three commercial vineyards (MOG, OSI, CAS) also in the Marche region, through application of a two-pronged approach based on point pattern analysis (PPA) and surface pattern analysis (SPA); and (iii) to define the relationships among the rate of emergence of symptomatic grapevines, the severity of BN, the rate of recovery, and the climatic conditions.

## Materials and methods

### Investigated areas

This study was carried out in a pilot vineyard cultivated with ‘Chardonnay’ (MOV), and in three commercial ‘Chardonnay’ vineyards (MOG, OSI, CAS), with all located within the Marche region. Supplementary Table S1 gives the details of their main features (i.e., location, cultivated area, geographic coordinates, altitude, rootstock, plant spacing, trellis system, year of planting), their surrounding crops/vegetation, their soil and pest management, and the main agronomical practices followed. For OSI and CAS vineyards, IPM practices were adopted, after an accurate monitoring of symptoms. In particular, copper-based formulations were used to control grapevine downy mildew, powdery sulphur for powdery mildew, and *Bacillus thuringiensis* was used for *Lobesia botrana*, while weeds were managed through mechanical tools. For the pilot vineyard MOV, data about production (weight, °Brix) from 2009 to 2015, were provided by the owner.

The main climatic data (i.e., mean monthly temperature, cumulative monthly rainfall) of the areas for the relevant years of investigation were provided by the local ASSAM weather stations (*Agenzia Servizi Settore Agroalimentare Marche*; Marche Region, Ancona, Italy). These data were used to calculate annual and seasonal (i.e., spring, summer, autumn, winter) mean temperatures and rainfall for each vineyard, and to define climatic anomalies respect to regional data collected from 1981 to 2010.

### Disease assessment and sanitary status of the vineyards

Visual inspections were carried out for MOV from 2009 to 2015 in mid-September, and similarly in MOG, CAS, OSI from 2013 to 2015. During the surveys, the positions of symptomatic grapevines were recorded on a two-dimensional map, and the disease severity was evaluated according to an empirical scale^6^. Overall, in the period 2013–2015, one hundred seventy BN-symptomatic, 95 recovered and 115 healthy grapevines were randomly sampled in the surveyed vineyards to assess the presence of ‘*Ca*. P. solani’. The total DNA was extracted according to the protocol suggested by Angelini *et al*.^33^ then the biomolecular assays were based on the use in nested PCR with fStol/rStol, according to Maixner *et al*.^34^. The phytosanitary status was recorded for each vineyard. The data recorded were used to calculate the following:

1 $$\begin{array}{c}{\rm{BN}}\,{\rm{infection}}\,{\rm{rate}}\,({\rm{per}}\,{\rm{year}};\,{\rm{total}}\,{\rm{symptomatic}}\,{\rm{grapevines}};\,{tS})\,=\\ \,{\rm{Number}}\,{\rm{of}}\,{\rm{symptomatic}}\,{\rm{grapevines}}/{\rm{total}}\,{\rm{number}}\,{\rm{of}}\,{\rm{grapevines}},\end{array}$$

2 $$\begin{array}{c}{\rm{Annual}}\,{\rm{new}}\,{\rm{symptomatic}}\,{\rm{grapevines}}\,({aNS})\,=\\ \,{\rm{Number}}\,{\rm{of}}\,{\rm{new}}\,{\rm{symptomatic}}\,{\rm{grapevines}},\,{\rm{recovered}}\,{\rm{or}}\,{\rm{asymptomatic}}\,{\rm{the}}\,{\rm{previous}}\,{\rm{year}},\end{array}$$

3 $$\begin{array}{c}{\rm{Annual}}\,{\rm{new}}\,{\rm{symptomatic}}\,{\rm{grapevines}},\,{\rm{never}}\,{\rm{before}}\,({aNnbS})\,=\\ \,{\rm{Number}}\,{\rm{of}}\,{\rm{new}}\,{\rm{symptomatic}}\,{\rm{grapevines}},\,{\rm{which}}\,{\rm{in}}\,{\rm{previous}}\,{\rm{years}}\,{\rm{were}}\,{\rm{always}}\,{\rm{asymptomatic}},\end{array}$$

4 $$\begin{array}{c}{\rm{Symptom}}\,{\rm{persistence}}\,=\\ \,{\rm{Number}}\,{\rm{of}}\,{\rm{symptomatic}}\,{\rm{grapevines}}\,{\rm{with}}\,{\rm{symptoms}}\,{\rm{for}}\,{\rm{two}}\,{\rm{consecutive}}\,{\rm{years}}\,{\rm{or}}\,{\rm{more}},\end{array}$$

5 $$\begin{array}{c}{\rm{Annual}}\,{\rm{rate}}\,{\rm{of}}\,{\rm{recovered}}\,{\rm{plants}}\,({aRec})\,=\\ \,{\rm{Number}}\,{\rm{of}}\,{\rm{recovered}}\,{\rm{plants}}\,{\rm{per}}\,{\rm{year}}\,{\rm{respect}}\,{\rm{to}}\,{\rm{symptomatic}}\,{\rm{vines}}\,{\rm{in}}\,{\rm{the}}\,{\rm{previous}}\,{\rm{year}},\end{array}$$

6 $$\begin{array}{c}{\rm{Temporary}}\,{\rm{recovered}}\,{\rm{plants}}\,({tRec})\,=\\ \,{\rm{Number}}\,{\rm{of}}\,{\rm{plants}}\,{\rm{recovered}}\,{\rm{for}}\,{\rm{one}}\,{\rm{or}}\,{\rm{two}}\,{\rm{consecutive}}\,{\rm{years}},\end{array}$$

7 $$\begin{array}{c}{\rm{Permanent}}\,{\rm{recovered}}\,{\rm{plants}}\,({pRec})\,=\\ \,{\rm{Number}}\,{\rm{of}}\,{\rm{plants}}\,{\rm{recovered}}\,{\rm{for}}\,{\rm{three}}\,{\rm{to}}\,{\rm{five}}\,{\rm{consecutive}}\,{\rm{years}},\end{array}$$

8 $$\begin{array}{c}{\rm{Rate}}\,{\rm{of}}\,{\rm{recovered}}\,{\rm{plants}}\,{\rm{that}}\,{\rm{showed}}\,{\rm{symptoms}}\,{\rm{again}}\,({SRec})\,=\\ \,{\rm{Number}}\,{\rm{of}}\,{\rm{symptomatic}}\,{\rm{plants}}\,{\rm{that}}\,{\rm{were}}\,{\rm{temporary}}\,({StRec})\,{\rm{or}}\,{\rm{permanently}}\,{\rm{recovered}}\,({SpRec})\,{\rm{in}}\,{\rm{previous}}\,{\rm{years}},\end{array}$$

9 $$\begin{array}{c}{\rm{Recovery}}\,{\rm{rate}}\,{\rm{of}}\,{\rm{symptomatic}}\,{\rm{plants}}\,=\\ \,{\rm{Number}}\,{\rm{of}}\,{\rm{recovered}}\,{\rm{grapevines}}\,{\rm{that}}\,{\rm{were}}\,{\rm{symptomatic}}\,{\rm{for}}\,{\rm{one}}\,{\rm{to}}\,{\rm{six}}\,{\rm{years}}\,{\rm{previously}},\end{array}$$

10 $$\begin{array}{c}{\rm{Total}}\,{\rm{asymptomatic}}\,{\rm{plants}}\,{\rm{per}}\,{\rm{year}}\,({tA})\,=\\ \,{\rm{Number}}\,{\rm{of}}\,{\rm{asymptomatic}}\,{\rm{grapevines}}\,{\rm{that}}\,{\rm{were}}\,{\rm{never}}\,{\rm{symptomatic}}\,{\rm{in}}\,{\rm{previous}}\,{\rm{years}},\end{array}$$

11 $${\rm{Mean}}\,{\rm{disease}}\,{\rm{severity}}\,({S})=\Sigma (c\times f)/n,$$

where *c* is the value of the severity class, *f* is the frequency in the severity class, and *n* is the number of symptomatic grapevines^35^.

The mean disease severity was calculated for the always symptomatic grapevines (*AS*), the annual new symptomatic grapevines (*aNS*), and the recovered plants that showed again symptoms (*SRec*), for each year of survey. These data were statistically analysed, applying Turkey’s HSD test for multiple comparison of means, at P ≤ 0.05 using R software (ver. 3.1.2, R Development Core Team) equipped with ‘car’ package. The same approach, method and software was used to statistically analyse the data according to the period of recovery (one to five years) before showing again symptoms, and recovery rate of plants after a different period of symptom persistence (one to six years).

### Spatial and temporal analysis of Bois noir in the vineyards

The spatially explicit datasets in the present study were organised and managed in a GIS environment. For each plant, the following attributes were collected: study site (MOV, MOG, CAS, OSI), geographic position (latitude, longitude), phytosanitary status (S, symptomatic; A, asymptomatic; R, recovered) and disease level (0–4, asymptomatic to seriously affected).

For the MOV site only, a natural neighbour spatial interpolation method was adopted to obtain a map of the disease level for the period of 2011–2015. This simple geostatistical method is available in the ArcMap software, and it provided the surface data (spatial resolution, 0.2 m) from a point dataset.

The index of aggregation (nearest neighbour index; NNI) was calculated in the ArcMap software, through the mean nearest neighbour tool (Spatial Statistics), to obtain a unique value for each site and year^36^. The NNI was then used in Pearson’s correlation analysis (Pearson’s r), against the climatic and main epidemiological data. PPA methods were applied to define the spatial patterns of the symptomatic plants within the four vineyards (CAS, MOG, MOV, OSI), and the spatial correlations of the disease level variable. The pair-correlation univariate function (*g*(*r*)), a second-order statistic that provides information at multiple spatial scales^37^, was calculated for each site. The pair-correlation function is non-cumulative and uses only points separated by a certain distance *r*, allowing specific scales to be identified where significant point–point interactions occur, particularly at small spatial scales^38^. From a preliminary univariate PPA performed on each site separately, a similar overall spatial pattern was found in all sites. For this reason, we conducted the spatial analysis considering the four sites as pseudo-replications^38^, as we were interested in the mean spatial pattern of symptomatic plants within the four vineyards. The results of the four sites were thus combined in one mean graphic pair-correlation function, using the ‘combine replicates tool’, as included in the Programita software^37^. The univariate pattern of symptomatic plants was compared with the complete spatial randomness (CSR) null model, to allow for second-order effects^37^. The 95% confidence intervals for the univariate analyses were computed from 1000 Monte Carlo simulations, and the goodness-of-fit (GoF) tests for the null hypothesis (CSR) was performed^37,38^. The analysis was carried out only for the classes with more than 100 points, applying a 1 m lag distance and a maximum distance of 15 m using the grid-based software Programita, with a grid size of 1 m^2^ and a ring width of 5 m.

To determine the spatio-temporal patterns of the levels of disease, a normalized mark-correlation function, also known as r-mark-correlation function (*K*_*mm*_(*r*)), was used on three sites (CAS, MOV, OSI) for the 2013–2015 period^37,39^. This function is used to analyse the spatial relationships among points containing quantitative attributes (marks), and in this study the level of disease (classes 1 to 4) was the quantitative mark, with mark-correlation function used to test for positive or negative correlations between the values of the marks^37^. As a result of this analysis, positive correlation defined points that were closely located and with similar level of disease (mark), and negative correlation defined points that were closely located but with different level of disease (mark). All of the PPAs were computed using the Programita software^37^.

## Results

During the period from 2009 to 2015, the mean temperature recorded for these seven years was higher by ~1 °C with respect to the regional data (1981–2010). In particular, in the surveyed locations, the years 2009, 2011, 2012 and 2015 showed constistent anomalies for the mean temperatures during spring (from +0.9 °C, to +3.4 °C), summer (from +0.9 °C, to +3.3 °C), autumn (from +0.5 °C, to +2.3 °C), and winter (from +1 °C, to +3.3 °C). On the other hand, the annual precipitation (mm) that was recorded for the same period for these sites surveyed in the Marche region was generally higher than the mean of regional climatic data (1981–2010).

### Sanitary status of the vineyards

Independent of the location, these symptomatic ‘Chardonnay’ grapevines in the vineyards surveyed showed light BN symptoms at the end of June, which progressively became more severe during the season, to reach their apex in the middle of September. These symptoms were characterised by discoloured yellowing leaves, leaf rolling that often resulted in an angular shape, dried out flowers, bunches on plants that became brown and shrivelled, with numerous small pustules seen along diseased branches (Figure S1). In 153 out of 170 (90%) of the symptomatic samples analysed in the nested PCR with the primers fStol/rStol, a specific amplicon of ~500 bp of ‘*Ca*. P. solani’ was obtained. No amplification was obtained in the recovered and asymptomatic samples collected (data not shown).

The data collected in September during the period 2009 to 2015 for the pilot vineyard MOV, and during the period 2013 to 2015 for the further vineyards MOG, OSI and CAS, were summarised to define how the sanitary status of these vineyards changed over time. The sanitary status was determined from the equilibrium rates of the asymptomatic, symptomatic and recovered grapevines.

In the MOV vineyard, a bell-shaped curve of total symptomatic plants (*tS*) was recorded over the 7 years, with the highest level reached in 2011, where there were 814 symptomatic plants, of a total of 1629 plants (50%); the lowest number was seen for 2015 (9.2%) (Table 1). The annual rates of new symptomatic plants (*aNS*) were around 50% of the total symptomatic plants (Table 1). Most of *aNS* were plants that were ‘never before symptomatic’ (*aNnbS*) in the previous years (491 in 2009, 320 in 2010, 421 in 2011) (data not shown), while from 2012 there was a large reduction (73 plants) that continued up to 2015, when there were only 10 never before symptomatic plants (data not shown). Over these seven years, there were only 5 plants that were always symptomatic for the seven consecutive years, 9 for six consecutive years, 30 for five consecutive years, 89 for four consecutive years, and 318 for three consecutive years (Table 1). It is worth noting that after seven years of assessment in the pilot vineyard MOV, there were 237 plants (14.5%) that had never shown any BN symptoms (Table 1).

**Table 1:** Phytosanitary status in the pilot vineyard MOV, as recorded from 2009 to 2015.

Year of survey	Plants											
	Asymptomatic	Symptomatic								Recovered		
	Total (*tA*) [n(%)]	Total (*tS*) [n(%)]	Annual new (*aNS*) [n]	Symptom persistence in year [n]						Annual (*aRec*) [n (%)]	Temporary (*tRec*) [n]	Permanent (*pRec*) [n]
				2	3	4	5	6	7			
2009	1138 (69.9)	491 (30.1)	491									
2010	817 (50.1)	716 (43.9)	320	396						96 (19.5)	96	
2011	395 (24.2)	814 (50.0)	470	151	193					372 (51.9)	420	
2012	324 (19.9)	422 (25.9)	162	152	44	64				549 (67.4)	840	43
2013	264 (16.2)	406 (24.9)	255	53	60	14	24			274 (64.9)	697	262
2014	244 (15.0)	205 (12.6)	131	47	6	10	3	8		328 (80.8)	556	624
2015	237 (14.5)	150 (9.2)	86	39	15	1	3	1	5	144 (70.2)	437	805
**Total**				**838**	**318**	**89**	**30**	**9**	**5**			

*tA*, total plants with no symptoms; *tS*, total plants with symptoms; *aNS*, plants with symptoms, that were recovered or asymptomatic in previous years; Symptom persistence, plants with symptoms for 2–7 consecutive years; Percentages in brackets calculated for all of the grapevines in the vineyard (N = 1629).

*aRec*, plants with symptoms in the previous year, that lost them each year (annual recovery rate); the percentage was calculated respect to the number of symptomatic plants in the previous year; *tRec*, plants with maintained recovery for 1 or 2 consecutive years; *pRec*, plants with maintained recovery for 3 to 5 consecutive years.

Considering the annual rates for the recovered plants (*aRec*), calculated respect to the symptomatic plants in the previous year, these generally increased from 19.5% (96/491) in 2010 to 80.8% (328/406) in 2014, then decreased at 70.2% (144/205) in 2015 (Table 1). Starting from 2012, the number of plants that recovered annually was usually higher than the number of plants that maintained the symptoms. In the last two years, there was a sharp increase in the permanently recovered plants (*pRec*) (Table 1).

The rate of recovered plants which showed again symptoms (*SRec*) was not statistically different considering the duration of previous recovered period (from one to five years) (Table 2). Also the recovery rates of plants was not statistically different according to the duration of persistence of symptoms (one to 6 years) (Table 3). A high mean of recovery rate (≥60%) was recorded for plants that showed symptoms for only one year and also for longer periods (up to five years) (Table 3).

**Table 2:** Rate of plants (*SRec*) showing again symptoms of BN after a period of recovery from one to five consecutive years in the pilot vineyard MOV.

Year of survey	Recovered plants that showed Bois noir symptoms again over the years [n(%)]				
	1	2	3	4	5
2011	48/96 (50.0)				
2012	84/372 (22.6)	5/48 (10.4)			
2013	119/549 (21.7)	64/291 (22.0)	6/43 (14.0)		
2014	34/274 (12.4)	49/423 (11.6)	24/225 (10.7)	4/37 (10.8)	
2015	12/328 (3.7)	28/228 (12.3)	27/389 (6.9)	6/202 (3.0)	3/33 (9.1)
Overall Mean (%)	**22.1 a**	**14.1 a**	**10.5 a**	**6.9 a**	**9.1 a**

These data were statistically analysed according to the period of recovery (1–5 years) before showing again symptoms, applying Turkey’s HSD test for multiple comparison of means, at P ≤ 0.05 using R software (ver. 3.1.2, R Development Core Team) equipped with ‘car’ package.

**Table 3:** Recovery rates of plants after a period of symptoms from one to six consecutive years in the pilot vineyard MOV.

Year of survey	Recovery rate of symptomatic plants according to persistence of symptoms over the years [n(%)]					
	1	2	3	4	5	6
2010	96/491 (19.5)					
2011	170/320 (53.1)	203/396 (51.3)				
2012	313/470 (66.6)	107/151 (70.9)	128/193 (66.3)			
2013	108/162 (66.7)	95/152 (62.5)	30/44 (68.2)	41/64 (64.0)		
2014	204/255(80.0)	48/53 (90.6)	49/60 (81.6)	11/14 (78.6)	16/24 (66.7)	
2015	92/131 (70.2)	34/47 (72.3)	4/6 (66.7)	7/10 (70.0)	2/3 (66.7)	3/8 (37.5)
Overall Mean (%)	**59.4 a**	**69.5 a**	**70.7 a**	**70.9 a**	**66.7 a**	**37.5 a**

These data were statistically analysed according to the year of symptom persistence (1–6 years), applying Turkey’s HSD test for multiple comparison of means, at P ≤ 0.05 using R software (ver. 3.1.2, R Development Core Team) equipped with ‘car’ package.

The disease severity, calculated for each of seven year of assessments, was generally higher in the always symptomatic plants (*AS*) compared to the new symptomatic plants (*aNS*), and to the recovered plants that showed symptoms again (*SRec*). In all cases though, there were no statistically significant differences seen year by year (Table S2).

Further data were collected in other three commercial vineyards (MOG, CAS and OSI) cultivated with Chardonnay too. The rate of symptomatic plants showed a decrease according to the year from 2013 to 2015 in the three vineyards (Table 4).

**Table 4:** Asymptomatic, symptomatic and recovered grapevines per year for the vineyards MOG, CAS and OSI (2013–2015).

Vineyard	Year of survey	Plants [n(%)]						
		Asymptomatic	Symptomatic				Recovered	
		total (tA) [n(%)]	Total (tS) [n(%)]	Annual new (*aNS*) [n]	Symptom persistence in years [n]		Annual *aRec* [n (%)]	R2Y [n]
					2	3		
MOG	2013	1015 (87.5)	145 (12.5)					
	2014	941 (81.1)	114 (9.8)	75	39		105 (72.4)	
	2015	919 (79.2)	54 (4.6)	33	6	15	93 (81.6)	94
CAS	2013	4866 (86.5)	760 (13.5)					
	2014	4523 (80.4)	736 (13.1)	344	392		367 (48.3)	
	2015	4288 (76.2)	579 (10.3)	302	100	177	453 (61.5)	306
OSI	2013	2171 (83.1)	443 (16.9)					
	2014	1972 (75.4)	413 (15.8)	202	211		229 (51.7)	
	2015	1911 (73.1)	220 (8.4)	88	47	85	286 (69.2)	197

*tA*, total plants with no symptoms; *tS*, total plants with symptoms; *aNS*, plants with symptoms that in the previous years were recovered or asymptomatic Symptom persistence, plants with symptoms for 2–3 consecutive years. Percentages in brackets are calculated for all of the plants in the vineyards (MOG, 1160; CAS, 5626; OSI, 2614). *aRec*, plants with symptoms in the previous year, that lost them each year (annual recovery rate); the percentage was calculated respect to the number of symptomatic plants in the previous year; *R2Y*, plants maintaining recovery for two consecutive years.

In the MOG vineyard, there was a reduction in the numbers of symptomatic plants, from 145 (12.5%) in 2013, to 54 (4.6%) in 2015, of the total of 1160 plants in the vineyard. The rate of annual recovered plants, calculated respect to the symptomatic plants in the previous year, were 72.4% (105/145) in 2014 and 81.6% (93/114) in 2015, and 89.5% (94/105) of the plants that recovered in 2014 and still remained in 2015 (Table 4).

In the CAS vineyard, there was again a reduction in the numbers of symptomatic plants, from 760 (13.5%) in 2013, to 579 (10.3%) in 2015, of the total of 5626 plants in the vineyard. The rates of recovered plants were 48.3% (367/760) in 2014 and 61.5% (453/736) in 2015, and 83.4% (306/367) of the plants that recovered in 2014 and still remained in 2015 (Table 5).

**Table 5:** Disease severity of symptomatic plants for the vineyards MOG, CAS and OSI (2014, 2015).

Vineyard	Year of survey	Disease severity of asymptomatic and symptomatic plants (1–4) [mean ± SE (n)]		
		Symptomatic		
		Always (*AS*)	Annual new (*aNS*)	Previously recovered (*SRec*)
MOG	2014	2.69 ± 0.16 (39) a	2.54 ± 0.08 (75) a	
	2015	2.40 ± 0.19 (15) a	2.49 ± 0.10 (33) a	2.44 ± 0.24 (11) a
CAS	2014	3.19 ± 0.04 (392) a	2.79 ± 0.05 (344) b	
	2015	3.01 ± 0.06 (177) a	2.54 ± 0.04 (302) c	2.71 ± 0.09 (65) b
OSI	2014	2.98 ± 0.06 (211) a	2.77 ± 0.07 (202) b	
	2015	2.85 ± 0.09 (85) a	2.67 ± 0.08 (88) b	2.93 ± 0.13 (28) a

These data were statistically analysed according to the year of survey and according to different categories of symptomatic plants (*AS*, *aNS*, *SRec*), applying Turkey’s HSD test for multiple comparison of means, at P ≤ 0.05 using R software (ver. 3.1.2, R Development Core Team) equipped with ‘car’ package. *AS*, plants always showing symptoms in previous years; *aNS*, plants with symptoms that were recovered or asymptomatic in previous years; *SRec*, plants with symptoms that in previous years were recovered. Numbers in brackets represent plants in each category.

In the OSI vineyard, there was a linear reduction of symptomatic plants, from 443 (16.9%) in 2013, to 220 (8.4%) in 2015, of the total of 2614 plants. The rates of recovered plants were 51.7% (229/443) in 2014 and 69.2% (286/413) in 2015, and 86% (197/229) of the plants that recovered in 2014 and still remained in 2015 (Table 4).

The mean disease severity calculated for always symptomatic plants (*AS*) was significantly higher (P < 0.05) respect to the annual new symptomatic plants (*aNS*) both in 2014 and 2015 for CAS and OSI vineyards (Table 5).

### Epidemiological patterns of Bois noir

**Figure 1:**
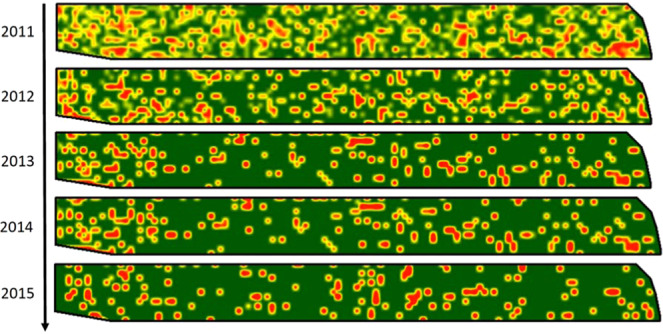
Disease level maps for site MOV obtained through natural neighbour interpolation of point datasets classified in five disease categories (0, asymptomatic to 4, severely affected). Raster maps (cell size, 0.2 m) calculated for 2011 to 2015.

The raster maps obtained through the natural neighbour spatial interpolation for the MOV site showed a general reduction in the pixels with intermediate disease values (1, 2; Fig. 1, yellow) for the 2011–2015 period. This trend resulted in a gradient from smoothed surfaces to more abrupt borders between severely symptomatic patches.

The mean aggregation (NNI) of the symptomatic plants for the 2013–2015 period ranged from low values at MOG and CAS (0.80, 0.85, respectively) to higher values at OSI and MOV (1.10, for both). MOG and MOV showed increasing trends for aggregation, whereas CAS showed a decrease, and OSI remained almost stable. At MOV, where the assessments were over a longer period (2009–2015), the NNI ranged from a maximum of 1.50 in 2011 to 1.10 during 2013 and 2014. The correlation analysis between this aggregation (NNI) of symptomatic plants and the climatic data showed no significant relationships, although, as expected, the density of the symptomatic plants was highly correlated to NNI.

**Figure 2:**
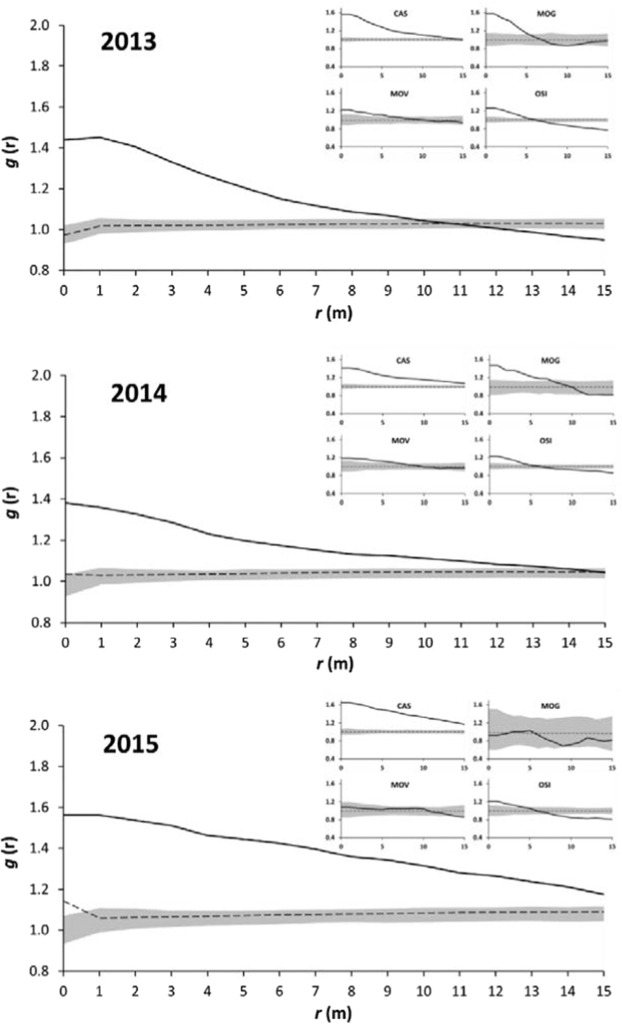
Univariate PPA of the overall symptomatic plants in the four vineyards of the Marche region using the combined replicates. Bold line, pair-correlation function *g*(*r*); dotted line, expected value under the null model (i.e., complete spatial randomness); shaded areas, non-significant (i.e., random) distributions representing 2.5th and 97.5th percentiles for *g*(*r*), from 999 Monte Carlo permutations. Insets: Results of each site separately.

The overall spatial patterns of the symptomatic plants were assessed according to univariate PPA under a complete spatial randomness null-model. This analysis showed a significant clustered distribution up to 10 m in 2013, then 13 m in 2014, and >15 m in 2015 (GoF: p ≤ 0.01, for all) (Fig. 2). This underlying clustered pattern was valid for all of the vineyards studied except for MOG and MOV in 2015, which showed random patterns (Fig. 2, inset). This situation is given as the mean overall pattern from the use of the ‘combine replicates’ technique, and it showed a general increase in the scale of aggregation between the symptomatic plants.

The spatio-temporal patterns of the levels of disease were analysed according to univariate normalised mark-correlation functions. These showed significant positive correlations for the 2013–2015 period for CAS only, within the range of the 1–3 m scale (GoF: p ≤ 0.002) (Table 6). This positive correlation means that neighbouring plants tended to have similar levels of disease.

**Table 6:** Univariate normalised mark-correlation function of the magnitude of symptoms at the CAS, MOV and OSI vineyards for the 2013–2015 period.

Site	Year	Scale (m)																Goodness of fit
		0	1	2	3	4	5	6	7	8	9	10	11	12	13	14	15	(p-value)
CAS	2013	○	●	●	●	○	○	○	○	●	○	●	○	○	○	○	○	**0.001**
	2014	○	●	●	●	●	●	●	○	●	○	●	●	●	●	○	○	**0.001**
	2015	○	●	●	●	○	●	○	○	○	○	○	○	○	○	○	○	**0.002**
MOV	2013	○	○	○	○	○	○	○	○	○	○	○	○	○	○	○	○	0.052
	2014	○	○	○	○	○	○	○	○	○	○	○	○	○	○	○	○	0.151
	2015	○	○	○	○	○	○	○	○	○	○	○	○	○	○	○	○	0.895
OSI	2013	○	○	○	○	○	○	○	○	○	○	○	○	○	○	○	○	0.523
	2014	○	○	○	○	○	○	○	○	○	■	○	○	○	○	○	○	**0.019**
	2015	○	○	○	○	○	○	○	○	○	○	○	○	○	○	○	○	0.648

Positive and negative significant correlations are indicated by filled circles and squares, respectively. Independent marking or randomness is indicated by open circles.

## Discussion

In the pedoclimatic conditions of central-eastern Italy, grapevines infected by ‘*Ca*. P. solani’ generally start to show symptoms in mid-June, according to the seasonal trend. Endeshaw *et al*.^10^ described the progressive development of symptoms in ‘Chardonnay’ grapevines, along with the changes in their physiological parameters (e.g., net photosynthesis, stomatal conductance, transpiration), from before the appearance of symptoms to the harvest (August-September)^10^. In these months, grapevines are considered to be diseased when at least three of the typical parameters are observed, from among the following: (i) shrivelled berries or clusters; (ii) lack of lignification in shoots; (iii) black pustules on the shoots; (iv) partial or total leaf yellowing and/or downward leaf curling; and (v) leaf blade fall, with the petiole still attached to the plant, as a late symptom. In this way, the picture of the sanitary status from the visual assessment is more reliable, because the grapevines, showing unambiguous symptoms, have generally high phytoplasma titre, thus there is a robust correlation between symptomatic plants and detection of ‘*Ca*. P. solani’ by molecular tools^6,40^. The different locations assessed in the present study (MOV, MOG, Montalto delle Marche; OSI, Osimo; CAS, Castelplanio) showed small differences in the temperatures recorded. However, climatic parameters were not directly correlated with the NNI index, calculated for the symptomatic plants, and epidemiological parameters determined (e.g., rate of newly infected and recovered plants, disease severity), having same pedoclimatic conditions. On the other hand, Panassiti *et al*.^11^ using a Bayesian model demonstrated that the presence of the BN disease increased with the presence of the vectors (*H. obsoletus*) and it was correlated with environmental conditions (altitudes, mean annual temperature), as well as with grapevine cultivars. This dataset for the BN epidemic presented and analysed here is of particular importance because of the density of the measurements (as individual plants), the tracking of the symptomatic and recovered plants over time and space, and the length of time over which these measurements were taken. Indeed, a similar approach was also followed recently to describe FD epidemiology in a vineyard of cv ‘Barbera’ in Piedmont^41^.

In the pilot vineyard MOV, where the visual assessments were carried out annually in September from 2009 to 2015, there were two severe outbreaks of BN in 2010 and 2011, with peak incidence levels of about 44% and 50%, respectively. The rate of infection then progressively decreased, to reach about 9% in 2015.

The rates of annual new infections here were high, as generally 50% of the total symptomatic plants. These data suggest the intense activity of the BN vector *H. obsoletus*, as demonstrated by research carried out in vineyards of Marche region^42^. Although the vectors usually feed on weeds in vineyards, in the absence of these, they are stimulated to move onto the grapevines more frequently. Panassiti *et al*.^43^ demonstrated that the low density of the host plant *U. dioica* and high vector abundance increase pathogen occurrence. In our situation, the weed management inside the vineyard chosen by the growers saw weed elimination in May, when the grapevines can suffer from their competition. This then directed the vectors towards feeding on the grapevines, which had become the only host plants available for these insects^11,44^.

Indeed, such cultural practices against these host plants, such as weeding and herbicide application, should not be carried out during the flight period of *H. obsoletus*, to prevent movement of the vectors towards the grapevines^45,46^.

For the persistence of symptoms, it is worth noting that only a few of the grapevines persistently showed symptoms, in agreement with Rott *et al*.^47^; hence, the sanitary status of vineyards infected by BN can be interpreted as a dynamic situation. After the two BN epidemic years (2010, 2011), in 2012 there was initially an increment in symptom remission that was related to the temporary recovered grapevines (*tRec*; with 1–2 years of recovery). In the following years, the trend towards recovered plants was confirmed, which reached a high rate (>75%) for these grapevines (*pRec*; recovery of>3 years), of around 50%. The total production of the vineyards was also dramatically reduced around the epidemic years, but then increased for the last 3 years, to reach acceptable grape quantity and quality (Table S3).

The persistence of symptoms was not related with the rate of recovery here. The chance for these ‘Chardonnay’ grapevines to recover was significantly higher independent of the persistence of symptoms. It appears not profitable the replacement of plants affected by BN when the recovery is the most course of the disease^4^. Not only for BN, high rates of natural recovery from FD have been reported for ‘Prosecco’ grapevines, as well as for ‘Chardonnay’ grapevines^48^.

The duration of the previous recovery period also did not influence significantly the overall average of the rate of grapevines that showed disease symptoms again after undergoing recovery. However, several studies theorized the physiological basis of recovery. Cytological and biochemical analyses revealed that in the recovered plants, the genes encoding chalcone synthase, phenylalanine ammonia-lyase, and class III chitinase appear to be consistently involved in the recovery phenomenon, with their gene expression not affected by plant phenology^21^. Specifically, as well as in apple and apricot, it has been shown in grapevine that recovery coincides with the accumulation of hydrogen peroxide in the sieve tubes, which often induces increased resistance^26^. In addition, there is abnormal callus accumulation, along with the associated proteins, due to the overexpression of the genes coding for callus synthase and protein P, as has been seen for recovered apple plants^49,50^. This defined the hypothesis that the recovered plants can develop resistance mechanisms dependent on Ca^2+^ signalling activity. Generally, if we consider the history of the grapevines in the present study, the risk of symptom expression in those showing recovery for 3–5 years was lower than in the always healthy grapevines, but without any statistical significance.

The space-time PPA carried out here in the pilot vineyard MOV over multiple years (2011–2015) allowed statistical description of the BN progression and regression dynamics. Considering the distribution of the grapevines in the vineyard according to the severity of BN symptoms, the maps for MOV obtained through natural neighbour spatial interpolation provide graphical visualisation of the grapevines that were more severely affected by BN. In 2011, these were positioned mainly along the borders of the vineyard, when the BN incidence was higher^51,52^. Mori *et al*.^53^ analysed the factors affecting the spread of “bois noir” disease, and they demonstrated that the incidence of border sides with nettle on vineyard surface was positively correlated to disease incidence in the vineyards with aggregate distribution of symptomatic plants. However, in our situation, we were not able to record nettle plants, but bindweed inside and around, as well as alfalfa plantations on the border. In a previous studies carried out in the vineyard MOV, we analysed most of symptomatic samples collected in 2011 and 2012 and most of them were molecularly characterized as ‘*Ca*. P. solani’ *tuf*-type b1 and only sporadic one were identified as *tuf*-type a^6,35^.

The general tendency to move from a diffuse mixed-severity pattern to a patchy spatial pattern would appear to be due to recovery of the less severely affected plants at the borders of the symptomatic patches. This geostatistical analysis allowed the hypothesis that the inoculum source was indeed outside the vineyard, and that it corresponded to the border where alfalfa (*Medicago sativa*) was grown. Moreover, when this approach was applied in the 2011–2015 period, it allowed evaluation of the progress of the disease in the field, to trace the epidemic history. This thus provides an understanding of dispersal routes of the pathogens, as well as the impact and efficiency of some of the control strategies and the role of the vectors, as has been reported for FD^41^. Indeed, the positions of the infected grapevines confirmed a natural source of inoculum and the activity of potential vectors in spreading BN in this vineyard. Recently, Quaglino *et al*.^54^ identified new ‘*Ca*. P. solani’ vectors to grapevines, and they hypothized, in Northen Italian pedoclimatic conditions, the spreading of phytoplasma to vineyard boarders through *H. obsoletus* and its further transmission within the vineyard by the feeding activity of the alternative vectors.

It is worth noting the gradual reduction in the disease levels in the fields from 2011 to 2015. The recovery in this vineyard over these years appears to be related to the plants that showed mild symptoms in the previous year, while most of the grapevines that showed more severe symptoms (i.e., classes 3, 4) remained symptomatic. The analysis of data showed that the mean disease severity recorded for the always symptomatic grapevines (*AS*) was generally higher, even if not statistically significant, than that of the annual new symptomatic vines (*aNS*). It was significantly evident in 2014 and 2015 in OSI and CAS vineyards. These data showed that in our pedoclimatic conditions the plants expressing symptoms from long time, showed systemic symptoms on more than 50% of canopy. On the other hand, new symptomatic plants generally showed localized symptoms on one or few shoots.

The PPA for the symptomatic plants in these four studied vineyards confirmed the visual trends through the geostatistical approach. Indeed, we observed an overall aggregated pattern at small (<10 m) spatial scales in 2013 that later became more evident at all spatial scales (0–15 m). The increased aggregation observed for the 2013 to 2015 period was significant for all of the sites studied. The overall spatial patterns of the symptomatic plants suggested that the length scale of the sampling is an important parameter in the complete spatial randomness validation, as the time at which the assessment is carried out. For vineyards OSI and CAS, a clustered distribution was recorded. The spatial arrangements of symptomatic plants in clusters was particularly evident for CAS, where there was positive spatial correlation between disease levels at the small spatial scales (1–3 m; mark-correlation function). This would appear to be due to the patchy distribution of *C. arvensis* in the area. In particular, the association of *U. dioica* and *C. arvensis* with BN symptomatic grapevines and insect vector captures, and their different distribution clustering within the examined vineyards, have been well documented in previous studies carried out in Europe under different pedoclimatic conditions^44,53,55,56^.

In the investigated vineyards, we did not observe an anisotropic pattern along the rows, as reported in a recent study by Maggi *et al*.^41^. This can be ascribed to the transmission of FD by the vector *Scaphoideus titanus* from grapevine to grapevine^57^, while in contrast, the behaviour of the vector(s) responsible of the spread of BN is not strongly affected by the linear pattern of the grapevines, but rather by the *C. arvensis* islands inside the vineyards^14,55,56^.

In conclusion, the data collected for a large number of single grapevine plants throughout a long period of monitoring, elaborated by this innovative approach based on PPA and SPA, allowed to highlight dynamics in BN incidence and severity fluctuation in time and space, to define the sanitary status of a vineyard as a dynamic balance between its symptomatic, recovered and healthy grapevines, and to trace and understand the dispersal routes of the pathogens. With this information supported by innovative^58^ and more sensitive molecular tools^59^, as well as molecular typing of *‘Ca*. P. solani’, it will be very useful for farmers for their decisions whether to replace or maintain a BN-infected grapevine.

## Supplementary information

Murolo et al., 2020 Scientific Reports - Supplementary information.docx.
