# Supplementary material for: Spatial pattern of Bois noir: case study of a delicate balance between disease progression and recovery
Source: Sci Rep. 2020 Jun 17;10:9801. doi: 10.1038/s41598-020-66210-7 (PMC7300118; doi:10.1038/s41598-020-66210-7)
Supplement: Supplementary file 1 — Murolo et al., 2020 Scientific Reports - Supplementary information.docx. [file 41598_2020_66210_MOESM1_ESM.docx]

**Spatial pattern of Bois noir: case study of a delicate balance between disease progression and recovery**

Sergio Murolo^1^, Matteo Garbarino^2^, Valeria Mancini^1^, Gianfranco Romanazzi^1^*

^1^ Department of Agricultural, Food and Environmental Science, Marche Polytechnic University, Ancona, Italy

^2^ Department of Agricultural, Forest and Food Sciences, University of Turin, Turin, Italy

^*^**Corresponding author: Gianfranco Romanazzi**

Department of Agricultural, Food and Environmental Science,

Marche Polytechnic University,

Via Brecce Bianche

60131 Ancona, Italy

Tel: +39-071-2204336

Fax: +39-071-220

**Table S1.** Main features of the MOV, MOG, OSI and CAS vineyards, which were all planted with cv. ‘Chardonnay’ on Kober 5BB (*V. berlandieri* × *V. riparia*) rootstock, using a single curtain trellis system and mineral fertilisation.

| **Vineyard feature** | **Vineyard** | | | |
| --- | --- | --- | --- | --- |
|  | **MOV** | **MOG** | **OSI** | **CAS** |
| Location | Montalto delle Marche | Montalto delle Marche | Osimo | Castelplanio |
| Province | Ascoli Piceno | Ascoli Piceno | Ancona | Ancona |
| Ownership | Petrelli farm | Petrelli farm | Moncaro winery | Moncaro winery |
| Vineyard area (ha) | 1.1 | 0.69 | 0.95 | 1.6 |
| Number of vines | 1629 | 1160 | 2614 | 5626 |
| Latitude | 43° 0'25.12"N | 43° 0'27.98"N | 43°29'0.81''N | 43°29'54.35''N |
| Longitude | 13°38'44.24"E | 13°38'32.71"E | 13°26'12.83''E | 13°5'3.53''E |
| Altitude (m a.s.l.) | 334 | 300 | 44 | 330 |
| Plant spacing (m) | 3.0 by 1.5 | 2.5 by 1.5 | 2.5 by 1.5 | 3.0 by 1.5 |
| Year of planting | 2006 | 2009 | 2002 | 2004 |
| Soil management | Tillage and chemical | Chemical | 4 mowing/year | 1 tillage, 3 mowing/year |
| Control of main fungal diseases | Chemical | Chemical | Integrated pest management | Integrated pest management |
| Surrounding crops/ vegetation | Vines, alfalfa, olive trees | Vines, alfalfa | Vines, wheat/ sunflower, alfalfa | Vines |

**Table S2.** Disease severity of the symptomatic plants in the pilot vineyard MOV, recorded from 2011 to 2015.

These data were statistically analysed according to the year of survey, applying Turkey’s HSD test for multiple comparison of means, at P ≤ 0.05 using R software (ver. 3.1.2, R Development Core Team) equipped with ‘car’ package. *AS*, plants always showing symptoms in previous years; *aNS*, plants with symptoms only in current year, while recovered or asymptomatic in previous year; *SRec*, plants showing symptoms that in previous years maintained recovery for 1 or 2 consecutive years (*StRec*) and for 3 to 5 consecutive years (*SpRec*). Numbers in brackets represent plants in each category.

| **Year of survey** | **Disease severity of symptomatic plants (1-4) [mean ±SE (n)]** | | | |
| --- | --- | --- | --- | --- |
|  | **Always (*AS*)** | **Annual new (*aNS*)** | **Recovered showing again symptoms (*SRec*)** | |
|  |  |  | **Temporary (*StRec*)** | **Permanent (*SpRec*)** |
| 2011 | 1.89 ±0.07 (193) a | 1.89 ±0.93 (470) a | 1.90 ±0.13 (48) a |  |
| 2012 | 2.58 ±0.12 (64) a | 2.33 ±1.08 (162) a | 2.35 ±0.10 (89) a |  |
| 2013 | 2.96 ±0.14 (24) a | 3.01 ±0.82 (255) a | 2.98 ±0.06 (183) a | 3.33 ±0.33 (6) a |
| 2014 | 2.88 ±0.23 (13) a | 2.72 ±0.73 (131) a | 2.71 ±0.08 (83) a | 2.76 ±0.14 (28) a |
| 2015 | 3.00 ±0.45 (7) a | 2.56 ±0.60 (86) a | 2.61 ±0.08 (40) a | 2.44 ±0.11 (36) a |

**Table S3. Productive data (weight and °Brix) recorded in the pilot vineyard MOV from 2009 up to 2015**

| **Date of harvest**  **(mm-dd-yy)** | **Productive data** | |
| --- | --- | --- |
|  | **100 Kg** | **°Brix** |
| 09-01-2009 | 30.00 | 19 |
| 08-29-2010 | 25.30 | 18.70 |
| 09-11-2011 | 22.70 | 18.50 |
| 09-02-2012 | 24.10 | 19.40 |
| 09-06-2013 | 28.90 | 20.70 |
| 09-09-2014 | 31.60 | 19.30 |
| 09-02-2015 | 33.40 | 20.10 |


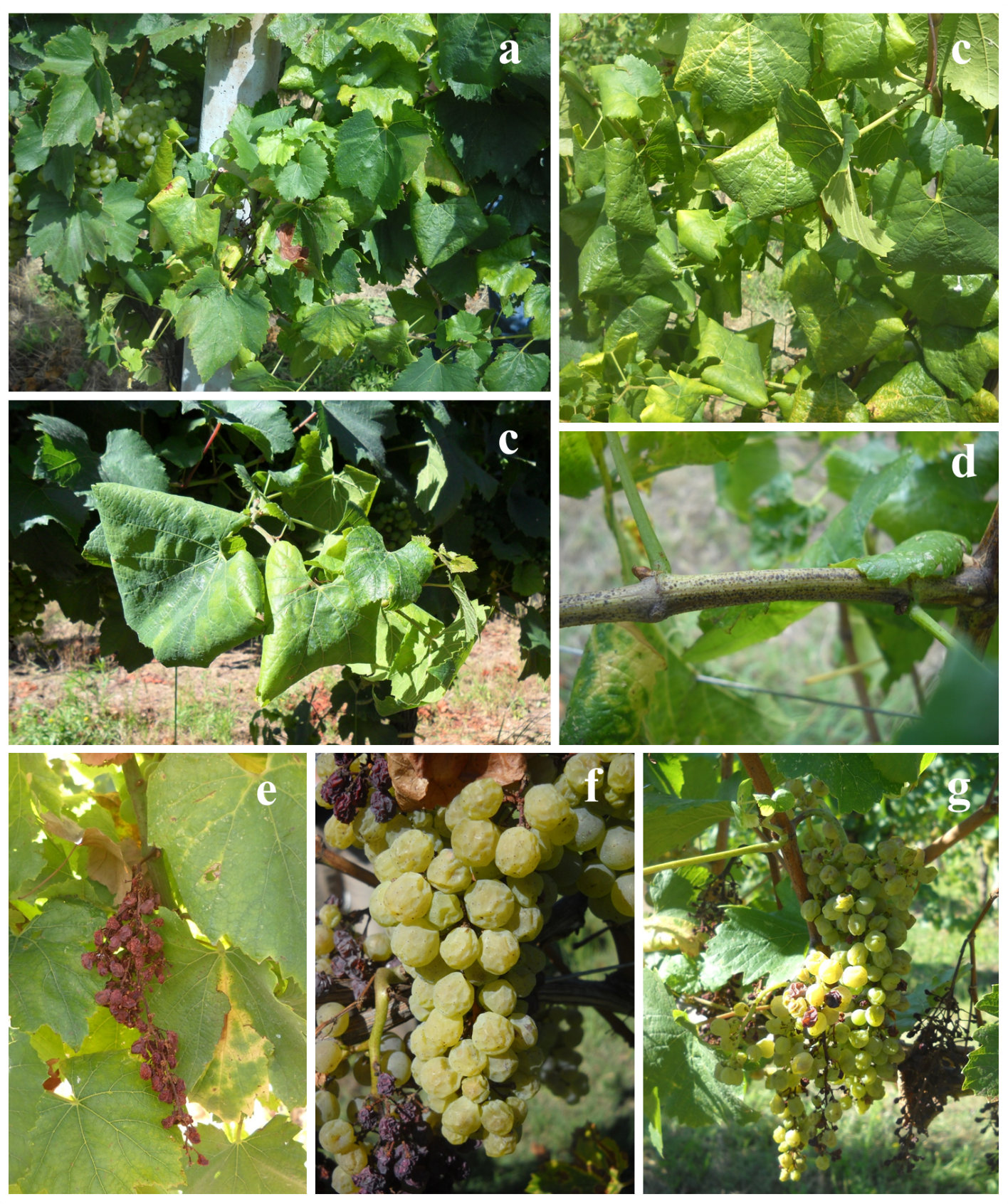


**Figure S1.** ‘Chardonnay’ grapevines infected by Bois noir. **(A-C)** Deformation and discoloration of leaf blades. **(D)** Necrotic areas on shoots of grapevine. **(E-G)** Partial or total dehydration of grape bunches.
